# Structural and Functional Properties Changes of β-Conglycinin Exposed to Hydroxyl Radical-Generating Systems

**DOI:** 10.3390/molecules22111893

**Published:** 2017-11-03

**Authors:** Jing Xu, Zijing Chen, Dong Han, Yangyang Li, Xiaotong Sun, Zhongjiang Wang, Hua Jin

**Affiliations:** 1 College of Science, Northeast Agricultural University, Harbin 150030, Heilongjiang, China; xujing@neau.edu.cn (J.X.); chenzijing_528@126.com (Z.C.); hanhahahhh@163.com (D.H.); ccwasdrdxnlyy@126.com (Y.L.); sunxt0523@163.com (X.S.); 2 College of Food Science, Northeast Agricultural University, Harbin 150030, Heilongjiang, China

**Keywords:** β-conglycinin, hydroxyl-radical, protein oxidation, emulsifying properties, allergenicity

## Abstract

The objective of the present study was to examine the structural and functional changes of β-conglycinin exposed to oxidizing radicals produced by FeCl_3_/H_2_O_2_/ascorbic acid hydroxyl radical-generating system (HRGS) for 3 h at room temperature. Increasing H_2_O_2_ concentrations resulted in a loss of histidine residues, lysine residues, and available lysine, which was accompanied by the formation of protein carbonyls and disulphide bonds (*p* < 0.05). Changes in secondary structure, surface hydrophobicity, and intrinsic fluorescence indicated that hydroxyl radicals had induced protein unfolding and conformational alterations. Results from SDS-PAGE implied that a small amount of protein cross-linkages produced by oxidative incubation. The emulsifying properties of β-conglycinin were gradually improved with the increasing extent of oxidation. The structural changes above contributed to the reduction of potential allergenicity of β-conglycinin, as verified by specific ELISA analysis. These results suggest that moderate oxidation could partially improve the protein functional properties and reduced the potential allergy of protein, providing guidance for effective use of moderately oxidized soy protein in the industry.

## 1. Introduction

Soybean protein, which accounts for about 40% of the dry weight of soybean seeds, is widely used as an important food ingredient in consumer diets due to its nutritional quality, functional characteristics, and quantity among seed legume proteins [1]. The functional properties of soy protein applied in the food industry have involved thickening, water absorption, emulsification, gelling, and foaming activities [2]. These properties vary with protein processing and storage conditions such as ionic strength, pH, temperature, and pressure, among others [3,4].

During processing and storage, soy protein is also vulnerable to oxidative attack [5]. Protein oxidation is the structural modification of a protein that is induced either directly by reactive oxygen species (ROS) or indirectly by secondary by-products generated from oxidative stress [6]. ROS mainly include free radicals (·OH, O_2_^•−^, etc.) and non-radical species (H_2_O_2_ and ROOH). The secondary by-products derived from lipid peroxidation primarily consist of lipid hydroperoxides and reactive aldehydes. Oxidative reactions of protein have been shown to induce a series of structural changes in protein backbones and amino acid residue side-chains, resulting in protein unfolding, backbone fragmentation, cross-linking, and conformational changes [7]. These structural changes contribute to deterioration or improvement in protein biological function and nutritional value, as well as physiochemical properties [8]. Oxidation that happens in meat and meat products has been extensively investigated. Some researchers have mimicked various kinds of lipid peroxidation-induced by-products to trigger protein oxidation, and found that oxidation has both positive and negative influences on the meat quality [9,10]. Hydroxyl radical-mediated protein oxidation has gained particular attention due to its highest rate constants for reactions with protein. The mechanism of the hydroxyl radical generating system (HRGS) is Fenton’s reaction, which generates hydroxyl radicals from H_2_O_2_ in the presence of Fe^2+^. Chanarat, Benjakul, and Xiong [11] reported that excessive oxidation via Fenton’s reaction induced obvious reduction in water-holding capacity and gel strength of myosin in tilapia proteins. Li et al. [12] reported that the myofibrillar proteins in common carp exhibited poor functional properties, such as emulsifying activity and emulsion stability, after oxidative incubation with HRGS. However, Liu et al. [13] demonstrated that the emulsifying properties of soy protein isolate were enhanced significantly with moderate oxidation by hydroxyl radicals, which has also been confirmed by Kong, Xiong, Cui, and Zhao [14]. Moreover, it turns out that oxidation has both positive and negative influences on the functional properties of proteins, depending on the different oxidants and oxidizing conditions (such as oxidizing concentration, time, and temperature) that are used [15,16].

Soy protein is not a single protein but is composed of several types of proteins with different characteristics. According to sedimentation coefficients, soybean protein can be classified into four fractions (2S, 7S, 11S and 15S) [17]. Among these fractions, the 7S fraction (mainly β-conglycinin), comprising approximately 20% of the total soy protein, is a trimer glycoprotein composed of the non-covalently linked α (72 kDa), α′ (76 kDa), and β (52 kDa) subunits [18]. β-conglycinin has been studied with the idea that it may explain most of soy’s beneficial properties and effects compared to other proteins, especially with regards to solubility, emulsifying properties, anti-atherosclerotic and anti-obesity effects [19,20]. However, β-conglycinin has also been shown to be one of the most important antigen proteins, which is highly involved in allergic reactions that disturb normal metabolism and interfere with digestion and absorption of nutrients [1]. This characteristic limits the application of β-conglycinin as well as soy protein in the food industry to some extent. However, there are very few practical applications in food processing that effectively reduce allergenicity [21]. In addition, little information is available regarding the relationship between the allergenicity and the structural characteristics of oxidized β-conglycinin.

Therefore, the objective of this study was to investigate structure and functional properties changes of β-conglycinin exposed to a simulated moderate oxidative environment with hydroxyl radical-generating system. The allergenicity of treated protein samples was measured, and their relationship with altered protein structures was discussed. These results are expected to provide guidance for the effective use of moderately oxidized soy protein in the industry and preventing the waste of oxidized protein materials.

## 2. Results and Discussion

### 2.1. Characterization of Oxidative Markers of Oxidized β-Conglycinin

The formation of protein carbonyl groups is considered to be one of the common markers for oxidative stress and is usually used to evaluate protein oxidative damage [22]. Effects of HRGS on total carbonyl groups of native and oxidized β-conglycinin are shown in Table 1. No significant difference (*p* > 0.05) in carbonyl content was observed when the H_2_O_2_ concentration was less than 1 mM. When oxidation was enhanced (1–15 mM), significant increases (*p* < 0.05) in carbonyl groups were observed up to a maximum value of 14.45 nmol/mg. Carbonyl groups are generated in proteins by ROS mainly through two pathways: (1) direct oxidative attacks on the side chains of susceptible amino acid residues, such as lysine, arginine, and proline residues, whose NH- or NH_2_- groups are vulnerable to ·OH oxidative attack and will transform into carbonyl groups [23]; (2) the cleavage of the protein backbone both by the α-amidation pathway occurring on α-carbon and β-scission reactions on the β-position [24]. Moreover, the initial carbon-centered radicals generated by these two pathways could react with ·OH at any available sites sequentially, forming a range of radicals and leading to further protein oxidation [25].

The increased protein carbonyl groups were also related to loss of histidine residues, lysine residues, and available lysine. Commonly, histidine and lysine residues refer to the remaining part of the structure in the formation of peptide bonds. Available lysine refers to an organic compound containing both amino and carboxyl group. Contents of histidine and lysine residues were determined by amino acid analysis and content of available lysine was determined by available lysine assay. In addition, these results are shown in Table 1. It was observed that the content of available lysine in oxidized β-conglycinin decreased significantly (*p* < 0.05) in an H_2_O_2_ concentration-dependent manner (Table 1). While histidine and lysine residues in modified β-conglycinin were almost remained the same with the native sample as 0.1 mmol/L H_2_O_2_ was employed. In addition, increasing the H_2_O_2_ concentration led to significant decreases (*p* < 0.05) in both histidine and lysine residue contents. It is noteworthy that β-conglycinin incubated with 15 mmol/L H_2_O_2_ exhibited 11%, 13% and 41% loss in the contents of histidine residues, lysine residues, and available lysine, respectively. Similar results were reported by Wu, Wu, and Hua [26] where the contents of available lysine and lysine residues of oxidized soy proteins incubated with 10 μmol/L acrolein decreased by 48% and 22%, respectively at 25 °C for 24 h. Since available lysine decreased more significantly than lysine residues in modified β-conglycinin, this indicates that available lysine is more susceptible to ·OH attack than lysine residues in β-conglycinin. The reaction of hydroxyl radicals with ε-NH_2_ groups of available lysine mainly contribute to the reduction of available lysine content. In addition, available lysine could also be consumed by interaction with existing carbonyls and carbonyl derivatives, thus forming protein cross-linkages [25].

The content of sulfydryl groups (SH) is another general indicator of protein oxidation. SH can be oxidized to either a reversible form (disulfide groups and sulfenic acid) or an irreversible form (sulfinic acid) in different oxidative environments [27]. Increasing concentrations of H_2_O_2_ resulted in a significant decrease in free SH (*p* < 0.05) and an increase in disulfide groups (*p* < 0.05) (Table 1). It was observed that incubation with 15 mM H_2_O_2_ caused an 89% loss of free SH and 12% increase in disulfide groups of β-conglycinin. This phenomenon indicated that the majority of the free SH transformed into disulphide bonds and other sulfur-containing oxidation products during oxidation. Similar results reported by Liu, Xiong, and Butterfield [28] showed a reduction of 42%, 38%, and 51% in free sulfhydryl of myofibrils, whey-protein isolates and soy-protein isolates, respectively after incubation with HRGS for 24 h. Among all amino acids, cysteine and methionine are the most susceptible to oxidative changes due to high reaction susceptibility of the sulfur group in those amino acids. Further, the oxidation of cysteine with radical oxidant can generate thiyl radicals, which can react with other thiol/thiolate to form disulfide; however, the major product from methionine oxidation is sulfoxide, which can be further oxidized to sulfone [25]. Due to the different main oxidation products of the two amino acids, the decrease in free sulfur group and an increase in disulfide groups could mainly attributed to the decrease or changes in cysteine residues induced by oxidation. Other studies demonstrated that the oxidative structural modification of proteins was accompanied by alterations in the redox state of cysteine and equilibrium constant of the thiol-disulfide interchange reaction, leading to changes in content and distribution of protein sulfhydryl and disulfide groups [29]. Furthermore, it has been pointed that cysteine residues and disulphide bonds have an important influence on the structure of proteins [30]. Therefore, changes in free SH and disulphide bonds could induce structural changes in oxidized β-conglycinin.

### 2.2. Changes in Secondary Structure of Oxidized β-Conglycinin

The secondary structures of native and oxidized β-conglycinin were determined using FTIR spectroscopy. The proportions of the α-helix, β-sheet, β-turn, and random coil for all the samples are listed in Table 2. Compared with the native β-conglycinin, all oxidative treated samples showed a gradual decrease in α-helix and β-sheet structures, and an increase in β-turn and random coil structures (*p* < 0.05). The secondary structure of protein depends both on the local sequence of amino acids and the interactions between different parts of the molecules [13]. The results indicated that oxidation had disrupted the intermolecular interactions, leading to secondary structure changes. In addition, it was observed that the α-helix contents decreased about 0.05% less than the loss of β-sheet (about 0.07%) in the 15 mM H_2_O_2_ treated sample. This may be due to the fact that α-helix has a compact structure with greater stability to attack oxidants, while the intermolecular hydrogen bonding of the β-sheet was destroyed significantly because of its less stable structure [31]. Furthermore, a decrease in the β-sheet component showed that the protein hydrophobic sites inside were exposed, corresponding to the enhanced surface hydrophobicity of the protein [32]. We also observed increases in β-turn and random coil structure, suggesting that protein unfolding, dissociation, and rearrangement of protein molecules occurred during oxidized incubation. Similar results were observed by Wu, Zhang, Kong, and Hua [8] where oxidation resulted in a gradual loss of α-helix with a concomitant decrease in β-sheet structure in soy protein. However, not all protein oxidation was accompanied by significant changes in the protein secondary structure, thus the different results may be attributed to the difference in oxidative environments and protein structure [26].

### 2.3. Changes in Surface Hydrophobicity and Intrinsic Fluorescence of Oxidized β-Conglycinin

**Figure 1:**
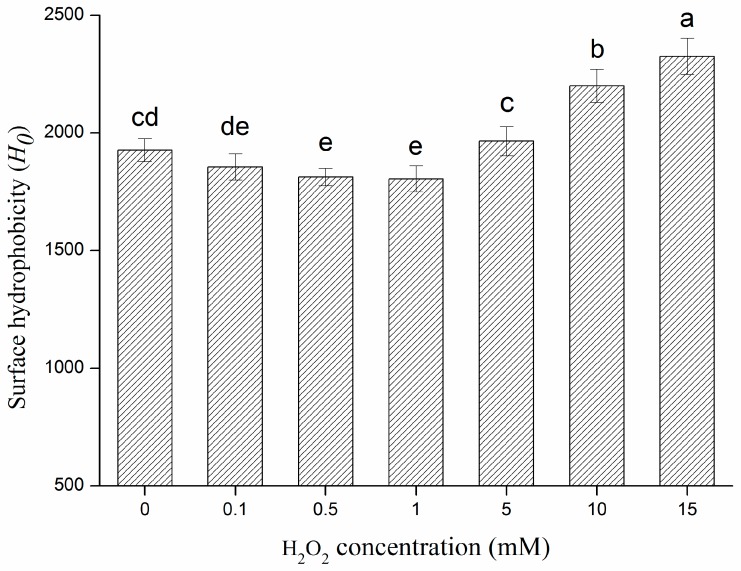
Effect of the increasing H_2_O_2_ concentration on the surface hydrophobicity of β–conglycinin. Columns with different letters (a–e) are significantly different (*p* < 0.05), which was concluded by SPSS software. In statistical analysis process, the effect of random error can be compared by probability value (*p*). If *p* < 0.05, it means that the effect of random error could be ignored and the difference of the columns in the Figure 1 are significative. Otherwise, columns with or within same letters (e and de, or c and cd, or cd and de) are not significantly different (*p* > 0.05). All data analysed in this figure are included in the supplementary information files.

Surface hydrophobicity was used as an acceptable marker for estimation of the level of unfolding in proteins [33]. Changes in surface hydrophobicity reflect changes in physical and chemical characteristics of protein structure [34]. Consequently, surface hydrophobicity was used as a probe to assess the conformational difference between native and modified β-conglycinin in this study. The surface hydrophobicity of β-conglycinin had a biphasic response to H_2_O_2_ compared with non-oxidized β-conglycinin, decreasing slightly initially (0–1 mM H_2_O_2_) and then increasing significantly (1–15 mM) (*p* < 0.05) (Figure 1). It has been found that the oxidation of surface-exposed hydrophobic residues and formation of hydrophilic groups (such as carbonyl groups) can be responsible for the decrease in protein surface hydrophobicity [35]. Surface-exposed hydrophobic residues (or side-chain residues) of protein are more susceptible to oxidation when the initiating oxidant is present in the bulk aqueous phase, and these processes often cause an increase in hydrophilicity [7,36]. Furthermore, the C-H bonds on aliphatic residues can transform into peroxide, alcohol, or carbonyl during oxidation and the oxidation of many aromatic residues can also lead to incorporation of polar groups such as hydroxyl-, peroxyl-, and nitro- [7]. On the other hand, oxidative modification resulted in protein unfolding, which was accompanied by the rearrangement of the protein structure and peptide bond cleavages, leading to the exposure of more interior hydrophobic groups and thus increasing surface hydrophobicity of the protein [26]. In conclusion, the mechanism of such changes in the current study can be explained by the balance between both the above factors. The observed decrease in surface hydrophobicity of oxidized β-conglycinin with lower H_2_O_2_ (0–1 mM) could be partially attributed to the formation of hydrophilic groups. However, protein unfolding and structure rearrangements caused by further oxidation (1–15 mM H_2_O_2_) dominated the subsequent process, leading to obvious increases in surface hydrophobicity. This result also implied that oxidative modification could result in alteration of protein structure, and that desirable structural changes in protein were attainable through regulating oxidant concentration.

**Figure 2:**
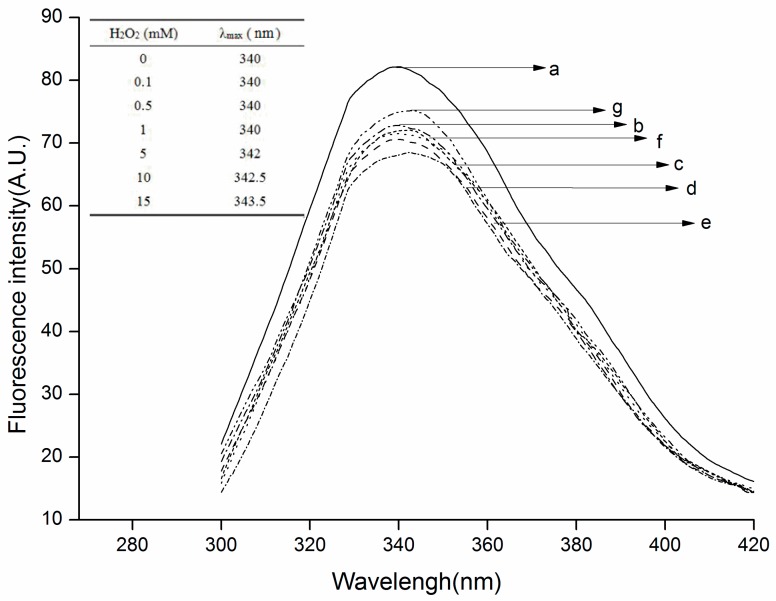
Intrinsic fluorescence spectras of the native and oxidized β–conglycinin. (The dissimilar letters (a–g) are corresponding to the seven different H_2_O_2_ concentration treated samples. a, 0 mmol/L H_2_O_2_; b, 0.1 mmol/L H_2_O_2_; c, 0.5 mmol/L H_2_O_2_; d, 1 mmol/L H_2_O_2_; e, 5 mmol/L H_2_O_2_; f, 10 mmol/L H_2_O_2_; g, 15 mmol/L H_2_O_2_). All data analysed in this figure are included in the supplementary information files.

Tryptophan residues (Trp) are a dominant fluorophore in proteins and are susceptible to forming a variety of oxidation products. Intrinsic fluorescence spectra of Trp residues in native and oxidized β-conglycinin were obtained to monitor the conformational changes and loss of Trp residues induced by hydroxyl radicals. As shown in Figure 2, it was observed that the fluorescence intensity of all the oxidized samples was lower than that of the native sample, and decreased initially (≤5 mmol/L H_2_O_2_) then increasing gradually (>5 mmol/L) with the fifth sample being the minimum point. The decrease in fluorescence intensity of all the oxidized samples could be attributed to the loss of Trp residues. Because they possess the lowest one-electron oxidation potentials among all the amino acid residues in the protein, Trp residues were initially converted into metastable tryptophan carbon-centered free radicals by ·OH, then yielded tryptophan peroxyl radicals to accelerate protein damage under aerobic conditions [37]. The increase in fluorescence intensity indicated that protein unfolding occurred during further hydroxyl radical-mediated oxidation, leading to exposure of native buried Trp residues. Also, a wavelength of the maximum emission (λ_max_) that is less than 330 nm would indicate that the Trp is assigned in a “nonpolar” environment, whereas a λ_max_ longer than 330 nm would imply that Trp is assigned in a “polar” environment. Thus, in this study, the Trp of all the samples were determined to be present in a “polar” environment [38]. In addition, exposure of β-conglycinin to increasing concentrations of H_2_O_2_ led to a red shift of maximum emission from 340 nm to 343.5 nm. The λ_max_ of tryptophan fluorescence is sensitive to its local environment. In general, red shifts of fluorescence maximum emission are attributed to the exposure of Trp residues to a hydrophilic environment while blue shifts are attributed to their exposure to a hydrophobic environment [16]. Under this condition, the results obtained in this work implied that the more Trp residues moved to the external side of oxidized β-conglycinin due to tertiary structure changes and protein unfolding.

### 2.4. Changes in Solubility of Oxidized β-Conglycinin

**Figure 3:**
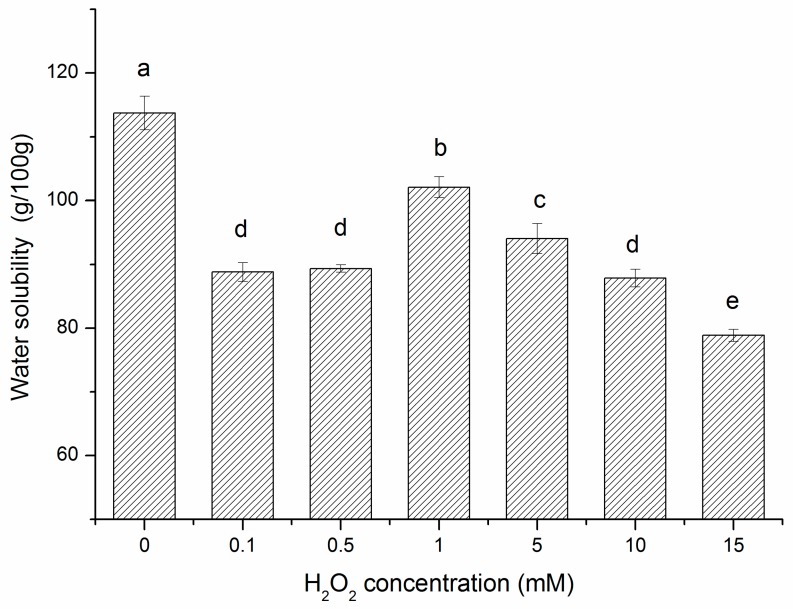
Solubility of β-conglycinin exposed to hydroxyl radical-generating systems with increasing concentrations of H_2_O_2_ for 3 h at room temperature. Columns with different letters (a–e) are significantly different (*p* < 0.05), which was concluded by SPSS software. In statistical analysis process, the effect of random error can be compared by probability value (*p*). If *p* < 0.05, it means that the effect of random error could be ignored and the difference of the columns in the Figure 3 are significative. Otherwise, columns with same letters are not significantly different (*p* > 0.05). All data analysed in this figure are included in the supplementary information files.

Water solubility is one of the important functional properties of β-conglycinin. The solubility of all the oxidative modified samples showed decreased compared with the control (Figure 3). Solubility is the result of interactions between protein and water through ion-dipole and dipole-dipole forces [14]. The loss of solubility indicated weakening of such forces due to the structural changes of β-conglycinin resulted from ·OH stressed. In addition, with the H_2_O_2_ concentration enhancement, the protein solubility showed increase first (0.1–1 mM) and then decrease gradually (1–15 mM) (*p* < 0.05). As in the case of lower H_2_O_2_ concentrations (0.1–1 mM), formation of hydrophilic groups (carbonyl groups) and the consumption of hydrophobic groups mainly contributed to the enhancement in solubility. Moreover, under higher H_2_O_2_ concentrations (5–15 mM), the formation of cross-linking structures above (disulfide groups) partially contributed to the decrease in the dissolution rate of protein. Combined with the results of surface hydrophobicity, exposure of more hydrophobic residues primarily led to further reduction in the dissolvability of oxidized β-conglycinin.

### 2.5. Changes in Emulsifying Properties of Oxidized β-Conglycinin

**Figure 4:**
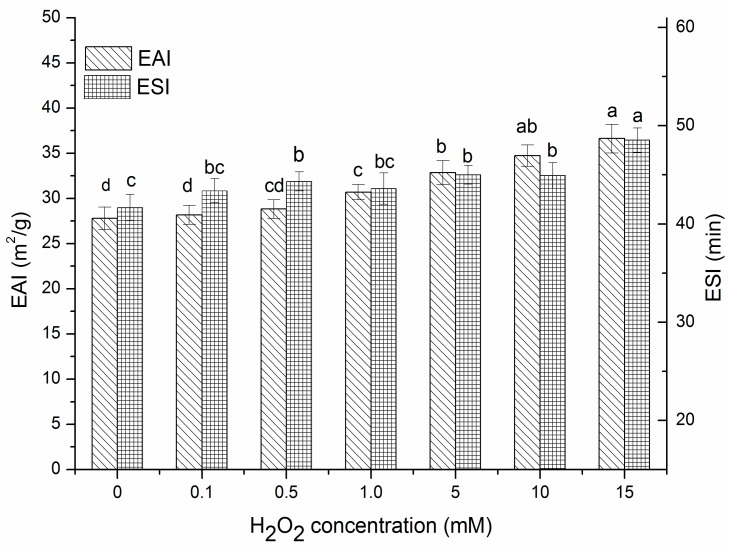
EAI and ESI of β-conglycinin exposed to hydroxyl radical-generating systems with increasing concentrations of H_2_O_2_ for 3 h at room temperature. Columns of EAI with different letters (a–d) are significantly different (*p* < 0.05), and columns of ESI with different letters (a–c) are significantly different (*p* < 0.05) which was concluded by SPSS software. In statistical analysis process, the effect of random error can be compared by probability value (*p*). If *p* < 0.05, it means that the effect of random error could be ignored and the difference of the columns in the Figure 4 are significative. Otherwise, columns with or within same letters (EAI: a and ab, or ab and b, or c and cd, or cd and d; ESI: b and bc, or bc and c) are not significantly different (*p* > 0.05). All data analysed in this figure are included in the supplementary information files.

Emulsifying properties refer to the oil-water interfacial area formed per unit weight of protein, characterizing the ability of a protein to stabilize emulsions. Emulsifying properties of proteins can be generally evaluated by the EAI and ESI [39]. In Figure 4, at lower H_2_O_2_ concentrations (0–0.5 mM), there were no obvious changes in EAI (*p* > 0.05), but at relatively higher H_2_O_2_ concentrations (1–15 mM), the EAI increased significantly (*p* < 0.05). ESI showed similar trends to EAI. Emulsifying properties depend both on the ability of protein adsorbed on the oil droplet surface and the protein intermolecular interaction, which is conducive to forming a stable absorption layer on the oil-water interface [40]. Consequently, the emulsifying ability of proteins is partly related to their surface hydrophobicity. Thus, the improvement in EAI and ESI at high concentrations of H_2_O_2_ could be attributed to exposure of hydrophobic groups, which allowed for more rapid adsorption of protein to the oil-water interface as well as stronger protein-protein interactions at interfaces [41]. In addition, moderate oxidation caused the partial unfolding of the protein structure thus increasing molecular flexibility [13]. In addition, flexible structures had been confirmed to diffuse more quickly to the oil-water interface, and easily rearrange to position their hydrophobic and hydrophilic groups in the oil and aqueous phase respectively [42]. All these consequences above may have contributed to obtaining better emulsifying properties.

### 2.6. Changes in the Subunits and Allergenicity of Oxidized β-Conglycinin

**Figure 5:**
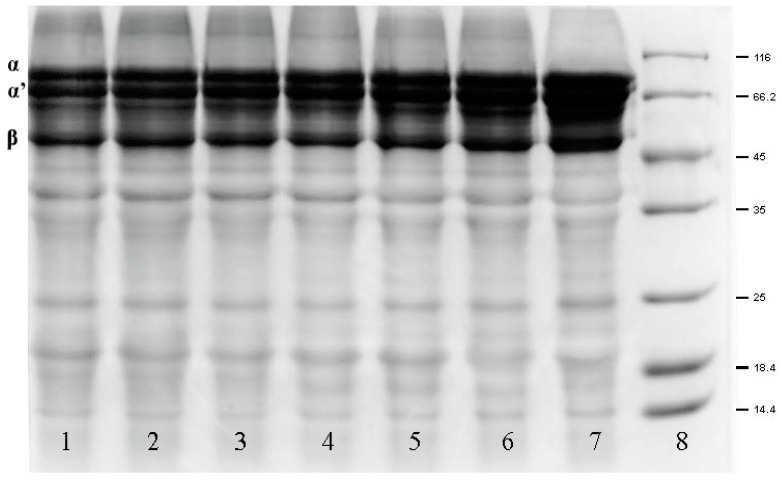
SDS–PAGE patterns of low molecular weight marker and oxidized β-conglycinin treated with different concentrations of H_2_O_2_ for 3 h at 20 °C. α, α’ and β bands showed the changes in three subunits of β-conglycinin. (Lane 1, 15 mmol/L H_2_O_2_; Lane 2, 10 mmol/L H_2_O_2_; Lane 3, 5 mmol/L H_2_O_2_; Lane 4, 1 mmol/LH_2_O_2_; Lane 5, 0.5 mmol/L H_2_O_2_; Lane 6, 0.1 mmol/L H_2_O_2_; Lane 7, β-conglycinin; Lane 8, low molecular weight marker). The molecular weights of markers are 14.4 kDa, 18.4 kDa, 25.0 kDa, 35.0 kDa, 45.0 kDa, 66.2 kDa, and 116.0 kDa, respectively.

SDS-PAGE was performed in order to monitor the subunit changes and protein cross-linking of β-conglycinin after oxidative modification [26]. Figure 5 shows the SDS-PAGE pattern of the seven samples (Lanes 1–7) and presents the characteristic bands for the three subunits of β-conglycinin (α, α’ and β). The pattern showed that the intensity and width of α, α’ and β bands of all oxidized β-conglycinin samples (Lanes 1–6) were reduced compared to those of the native sample (Lane 7). This indicated that cleavage of the peptide bond had occurred during the oxidation process, resulting in a loss of subunits, which was consistent with previous reports that the intensity of α, α’, and β bands had decreased with an increase in acrolein concentration from 1 to 10 μmol/L as demonstrated by soy protein SDS-PAGE [26]. These findings were also consistent with the results of the carbonyl content assay described earlier. In addition to the oxidation of amino acid side chains, carbonyls can be generated by peptide bond cleavages [43]. Therefore, the loss of the subunits was partly related to the formation of carbonyls. On the other hand, after oxidation, there were some smeared bands that appeared in the area above the subunit bands which corresponded to higher molecular weights (Lanes 1–6). These newly formed bands implied the generation of cross-linkings that could attributed to some active carbonyl-NH_2_ interactions, which was derived from further attacks on the free amino acids via the carbonyl groups [33]. The electrophoresis results showed that both fragmentation and cross-linking occurred in the subunits during oxidation. It is also known that α, α’, and β subunits of β-conglycinin are the major allergens in soy protein. The changes in the three subunits during oxidation may be propitious in reducing the allergenicity of β-conglycinin.

**Figure 6:**
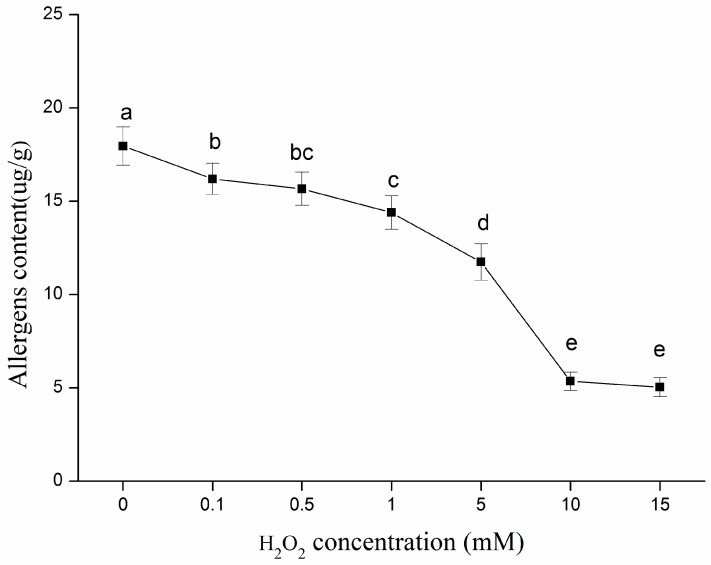
Changes on average contents of the allergens in native and oxidized β–conglycinin incubated with increasing concentrations of H_2_O_2_ in hydroxyl radicals–generating systems for 3 h at room temperature. Values with different letters (a–e) are significantly different (*p* < 0.05), which was concluded by SPSS software. In statistical analysis process, the effect of random error can be compared by probability value (*p*). If *p* < 0.05, it means that the effect of random error could be ignored and the difference of the values in the Figure 6 are significative. Otherwise, values with or within same letters (b and bc, or c and bc) are not significantly different (*p* > 0.05). All data analysed in this figure are included in the supplementary information files.

It is widely known that the ELISA process is based on the reaction between antigen and its relatively specific antibody. The immune-reactive components in β-conglycinin, which were used as the antigen in this method, were measured using this process [44]. The immune-reactive allergen contents of native and oxidized β-conglycinin would be able to reflect the potential allergenicity of these samples directly. As shown in Figure 6, the allergens in all the modified β-conglycinin samples were reduced notably (*p* < 0.05) compared with the native sample, in an H_2_O_2_ concentration-dependent manner. This result implied that the allergens of β-conglycinin were partially destroyed by oxidative incubation. The allergenicity of protein is associated with immunoglobulin E (IgE) recognition sites (so called IgE-binding epitopes) in protein antigens, thus the allergenic reaction can be attributed to IgE recognition of allergenic protein [45]. All the three subunits (α, α’, and β) of β-conglycinin are able to elicit IgE antibody in soybean-sensitive patients [46]. The epitope is the part of an antigen molecule to which an antibody attaches itself, and the IgE-binding epitopes can be divided into two types: linear (sequential) and conformational (discontinuous). The linear epitopes contain continuous amino acid sequences, while conformational epitopes are formed by spatially adjacent amino acids that are distantly located in the primary sequence of the proteins [47]. Therefore, combined with the alteration of the secondary and tertiary structures above, structural changes could result in the destruction of adjacent structures in amino acids, thereby weakening the ability of immune proteins to be recognized by antibodies, as has been confirmed by Song et al. [48]. Furthermore, the unfolding of β-conglycinin caused partial shielding of the antigenic epitopes so a protein conformation adjustment might destroy these epitopes. In addition, some active amino acid side chains presented in the epitopes were perhaps sensitive to attack by oxidants and formed carbonyls, which caused the denaturation of some epitopes, thus promoting the reduction of protein allergenicity [47]. Moreover, it has been confirmed that there is a negative correlation between the IgE binding of allergens and the protein carbonyl content [49]. Thus, the reduction of allergenicity could also be related to the increase in protein carbonyls in this study. Ultimately, the decrease in allergenic potential of β-conglycinin was mainly due to protein carbonylation and conformational changes during oxidation. It was also noteworthy that oxidative modification could not eliminate the allergenicity completely, which could be explained by protein unfolding leading to the exposure of some epitopes hidden inside the native protein.

## 3. Materials and Methods

### 3.1. Materials

Low temperature defatted soybean meal, a soybean byproduct prepared by oil immersion at a low temperature, was purchased from Harbin High-Tech Co. (Harbin, China). Ascorbic acid, ethylene diaminetetraacetic acid (EDTA), 5,5′-dithiobis (2-nitrobenzoic acid) (DTNB), and 2,4,6-trinitrobenzenesulfonic acid sol (TNBS) were purchased from Sigma Chemical Co. (St. Louis, MO, USA). The β-conglycinin ELISA kit was purchased from Crodibio Co. (Quanzhou, China). All chemicals and reagents obtained in this study were of analytical grade.

### 3.2. Preparation of β-Conglycinin

The β-conglycinin was prepared according to methods described in Wang, Wang, Handa, and Xu [32] and the protein content of β-conglycinin was determined by the Kjeldahl method as 80.32 ± 2.1% (*w*/*w*) with 6.25 used as a nitrogen to protein conversion factor. The moisture, fat, and other component contents of β-conglycinin were about 5.95 ± 0.4%, 1.62 ± 0.2% and 12.11%, respectively.

### 3.3. Preparation of Oxidized β-Conglycinin

Oxidized β-conglycinin was prepared according to methods described in Cui, Xiong, Kong, Zhao, and Liu [43] with a few modifications. The solutions of hydroxyl radical-generating systems (HRGS) contained 0.1 mM FeCl_3_, 0.1 mM ascorbic acid, and six levels of H_2_O_2_ (0.1, 0.5, 1, 5, 10, 15 mM) that were prepared primarily with 0.01 M phosphate buffer (pH 6.0). The β-conglycinin suspension (20 mg/mL) was then incubated and shaken in the hydroxyl radical-generating systems at 20 ± 1 °C for 3 h, and oxidation was terminated by adding BHA/Trolox/EDTA (1 mM each). The control sample was in phosphate buffer without the components of the hydroxyl radical-generating systems. The control and oxidized β-conglycinin samples were then freeze-dried and stored at 4 °C.

### 3.4. Measurement of Chemical and Structural Changes

#### 3.4.1. Protein Carbonyls

Protein carbonyls were quantified according to methods described in Liu, Lu, Han, Chen, and Kong [13] that were based on the reaction of 2,4-dinitrophenyl hydrazine (DNPH) with the carbonyl groups of native and oxidized β-conglycinin. The carbonyl contents were expressed as nmole per milligram of soluble protein with the molar extinction coefficient of 22,000 M^−1^ cm^−1^. The soluble protein concentration was measured using the biuret method.

#### 3.4.2. Amino Acid Analysis

Determination of histidine and lysine residue contents in native and oxidized β-conglycinin samples were carried out using hydrochloric acid hydrolysis method described in Uchida et al. [22] with modifications. 50 mg of each sample was placed in hydrolysis tube, added with 10 mL of 6 mol/L hydrochloric acid. After purged with nitrogen for 15 min, the samples were then incubated for 24 h at 110 °C. Then the solutions were transferred into a 50 mL volumetric flask and dilute with purified water to 50 mL. 1 mL of each solution was transferred into a 2 mL plastic sample tube, and then vacuum concentrated at 60 °C for 3–4 h to ensure the samples were evaporated completely. Concentrated samples were added with 1 mL of sample buffer (containing 2.7% sodium acetate trihydrate, 7.0% ethanol, 0.5% formic acid, 1.0% acetic acid and 2.95% trifluoroacetic acid (1:1 *v*/*v*)) and fully oscillated. Then the samples were filtered through a cellulose acetate membrane with a pore size of 0.45 µm and determined the histidine and lysine residues with an Aminosys A200 amino acid analyzer (Aminosys Co., Beijing, China).

#### 3.4.3. Available Lysine

Available lysine contents in native and oxidized β-conglycinin were determined using methods described in Benjakul and Morrissey [50], which used the reaction of the 2,4,6-trinitrobenzenesulfonic acid (TNBS) with available amino groups in protein. 125 µL of properly diluted samples was mixed with 2 mL of 0.2125 M, pH 8.2 phosphate buffer, and then 1 mL of 0.02% TNBS solution. The mixtures were placed in a water bath at 50 °C for 30 min in the dark and then incubated with 2 mL of 0.1 mol/L sodium sulfite to stop the reaction. The mixtures were then cooled down at room temperature for 15 min. The absorbance was measured at 420 nm, and pure l-lysine was used as a standard.

#### 3.4.4. Free Sulfhydryl and Disulfide Content

Free sulfhydryl and disulfide contents of native and oxidized β-conglycinin were determined by methods described in Huang, Hua, and Qiu [51]. Protein concentrations were measured using the biuret method, and the results were expressed as nmol of per milligram protein and calculated by using the extinction coefficient of 13,600 M^−1^ cm^−1^.The disulfide content of β-conglycinin was estimated by subtracting the free sulfhydryl from the total content of sulfhydryl and disulfide groups.

#### 3.4.5. Fourier Transform Infrared Spectra (FTIR)

FTIR spectra were scanned at a wavelength range of 4000 to 400 cm^−1^ by a FTIR spectrometer (Bruker Vertex 70, Bruker Optics, Ettlingen, Germany) at 25 °C. The values for the scan times, wave number precision, and resolution were 64, 0.01 cm^−1^, and 4 cm^−1^ respectively. The FTIR spectra data were calculated and analyzed by the algorithm of “Gaussian peak fitting” and the software “Peakfit Version 4.12”, which are commonly used for protein secondary structure analysis.

#### 3.4.6. Surface Hydrophobicity (*H*_0_)

The surface hydrophobicities of the native and oxidized β-conglycin samples were determined using ANS according to methods described in Huang, Hua, and Qiu [51] with some modifications. The protein suspension (2 mg/mL) was prepared with 0.01 M, pH 7.0 phosphate buffer and constantly stirred for 30 min using a magnetic stirring bar at room temperature and then centrifuged at 10,000 *g* for 15 min. The supernatant concentration was determined using the biuret method. Then serial dilutions of the supernatant were prepared with the same buffer at concentrations in the range of 0.005–0.5 mg/mL. 40 µL of ANS (8 mM, in the same buffer) was added to 4 mL of protein solution. Fluorescence intensity (FI) was measured at Ex 390 nm and Em 470 nm with an F-4500 Fluorophotometer (Hitachi, Tokyo, Japan). The protein hydrophobicity (*H*_0_) was the initial slope of the FI versus protein concentration.

#### 3.4.7. Fluorescence Measurement

The fluorescence spectra of native and oxidized β-conglycinin solutions (0.2 mg/mL) were obtained at 25 °C in 0.01 M phosphate buffer (pH 7.0) with an F-4500 Fluorophotometer (Hitachi, Tokyo, Japan) at Ex 290 nm, Em 300–420 nm with the slit of 5 nm and 10 nm/s of scanning speed according to Wang, Wang, Handa, and Xu [32].

#### 3.4.8. Electrophoresis

The native and oxidized β-conglycinin samples were performed by sodium dodecyl sulfate polyacrylamide gel electrophoresis (SDS-PAGE) as described by Laemmli [52] with modifications. 12% separation gel and 5% stacking gel were used in this experiment. Each line was loaded 10 μL of each sample then run at 80 V and 120 V constant voltages of stacking gel and separation gel, respectively.

### 3.5. Measurement of Functional Properties Changes

#### 3.5.1. Protein Solubility

The native and oxidized β-conglycinin samples (20 mg/mL) were dispersed in distilled water at pH 7.0 and constantly stirred for 1 h at room temperature, and then centrifuged at 10,000 rpm for 30 min at 25 °C. Protein solubility was determined from supernatants using the biuret method and expressed as grams of soluble protein/100 g protein in the sample.

#### 3.5.2. Determination of emulsifying Properties

Emulsifying properties were determined according to methods described in Pearce and Kinsella [53] with a few modifications. 5 mL soybean oil and 20 mL of protein solution (10 mg/mL) were mixed. The mixture was homogenized using a homogenizer (FJ200-SH, Shanghai Specimen and Model Factory, Shanghai, China) at a speed of 10,000 rpm for 2 min. An aliquot of the emulsion (20 μL) was pipetted from the bottom of the container at 0 and 10 min and then mixed with 5 mL of 0.1% SDS solution. The absorbance of the diluted solution was measured at 500 nm using a spectrophotometer (T6, Pgeneral, Beijing, China). The absorbances measured immediately (A_0_) and 10 min (A_10_) after emulsion formation were used to calculate the emulsifying activity index (EAI) and emulsion stability index (ESI) as follows:

(1) $$\mathrm{EAI}(m^{2}/g)=\frac{2\times 2.303\times A_{0}\times 250}{c\times 1\times (1-\varphi )\times 10,000}$$

(2) $$\mathrm{ESI}(\min)=\frac{A_{0}}{A_{0}-A_{10}}\times t$$

where *c* is the initial protein concentration (g/mL) before emulsification, l is the optical path (0.01 m), φ is the oil volume fraction (25%) used to form the emulsion, t is 10 min, and A_0_ and A_10_ are the absorbances of the diluted emulsions after 0 and 10 min of storage, respectively.

#### 3.5.3. Allergenicity Measurement

The contents of active allergenic protein in native and oxidized β-conglycinin samples were determined by a commercially specific ELISA as instructed by the manufacturer [54]. A room temperature balanced 96-well antibody microplate was prepared first. 50 μL of six levels of standard (11.25, 22.5, 45, 90, 180, 360 ng/mL) and 10 μL of properly diluted samples in 0.1 M phosphate buffer (pH 7.0) mixed with 40 μL of sample dilution were added to the standard and sample wells, respectively. Then 100 μL of HRP-conjugate reagent was added to each well and the microplate was sealed with a film, then incubated at 37 °C for 60 min, followed by washing of all the wells 5 times with washing solution after the reaction was completed. After adding Chromogen solution A (50 μL) and chromogen solution B (50 μL) to each well and protecting the microplate from the light at 37 °C for 15 min, the chromogenic reaction was terminated with 50 μL of stop solution which was added to all the reaction wells. Then the optical density (OD) value was measured at 450 nm using an automatic Tecan Genios (Spark 10 M, Tecan Co., Männedorf, Switzerland) plate reader. As instructed by the kit, the standard curve was obtained by plotting the OD values against standard sample (active allergen) concentrations and the standard equation was as follows: Y = 0.0053X + 0.1479, *R*^2^ = 0.992, where Y is the OD value at 450 nm, X is the standard sample concentrations (ng/mL). The concentration of active allergenic protein in the samples was calculated according to the standard equation and expressed as μg per gram protein.

### 3.6. Statistical Analysis

All experiments were performed in triplicate for each sample and the results are presented as mean ± standard. Statistical analysis was performed using SPSS (20.0) software (IBM, Armonk, NY, USA). The significant differences (*p* < 0.05) between means were identified using Duncan’s procedure.

## 4. Conclusions

Results from this investigation showed that moderate β-conglycinin oxidation induced the formation of protein carbonyls and disulfide groups with a concomitant reduction in histidine, lysine, and cysteine residues as well as available lysine and free sulfhydryl groups. Changes in the protein secondary structure, tertiary structure and SDS-PAGE analysis indicated the presence of protein unfolding and a small amount of protein cross-linking. This moderate oxidation led to a decrease in protein solubility and an improvement in emulsifying properties as well as reduction in the potential allergenicity of β-conglycinin. Further studies need to be performed to explore the over-oxidation effects and evaluate the toxicologic concerns of β-conglycinin.

## Figures and Tables

**Table 1:** Protein carbonyls, histidine residues, lysine residues, available lysine, sulfhydryl and disulfide groups contents of β–conglycinin exposed to hydroxyl radical–generating systems with increasing concentrations of H_2_O_2_ for 3 h at room temperature.

H_2_O_2_ (mmol/L)	Carbonyls (nmol/mg)	Histidine (g/100 g)	Lysine (g/100 g)	Available Lysine (g/100 g)	Free SH (nmol/mg)	Disulfide Groups (nmol/mg)
0	8.02 ± 0.29 ^e^	1.17 ± 0.01 ^a^	2.91 ± 0.02 ^a^	6.23 ± 0.08 ^a^	8.66 ± 0.04 ^a^	84.89 ± 2.28 ^bc^
0.1	8.14 ± 0.17 ^e^	1.16 ± 0.02 ^a^	2.89 ± 0.03 ^a^	5.96 ± 0.08 ^b^	8.58 ± 0.01 ^b^	81.42 ± 3.17 ^cd^
0.5	8.36 ± 0.27 ^e^	1.15 ± 0.01 ^ab^	2.82 ± 0.02 ^b^	5.44 ± 0.14 ^c^	7.42 ± 0.03 ^c^	79.25 ± 1.77 ^d^
1	9.18 ± 0.26 ^d^	1.13 ± 0.01 ^b^	2.79 ± 0.03 ^b^	5.06 ± 0.11 ^d^	5.18 ± 0.02 ^d^	88.44 ± 3.36 ^b^
5	10.95 ± 0.20 ^c^	1.10 ± 0.02 ^c^	2.71 ± 0.03 ^c^	4.29 ± 0.17 ^e^	1.36 ± 0.05 ^e^	89.13 ± 3.39 ^b^
10	13.73 ± 0.25 ^b^	1.08 ± 0.03 ^c^	2.62 ± 0.04 ^d^	4.09 ± 0.06 ^f^	1.01 ± 0.07 ^f^	95.18 ± 1.31 ^a^
15	14.45 ± 0.18 ^a^	1.04 ± 0.01 ^d^	2.54 ± 0.03 ^e^	3.68 ± 0.13 ^g^	0.95 ± 0.04 ^g^	96.24 ± 3.21 ^a^

All data points are represented as the mean of triplicate determinations ± standard errors. Values in the same column followed by different superscript letters (a–g) are significantly different (*p* < 0.05), which was concluded by SPSS software. In statistical analysis process, the effect of random error can be compared by probability value (*p*). If *p* < 0.05, it means that the effect of random error could be ignored and the difference of the value in the Table 1 are significative. Otherwise, the values in the same column followed by or within the same superscript letters are not significantly different (*p* > 0.05).

**Table 2:** Secondary structural content of native and oxidized β-conglycinin incubated with increasing concentrations of H_2_O_2_ in hydroxyl radicals–generating systems for 3 h at room temperature.

H_2_O_2_ (mM)	α-Helix (%)	β-Sheet (%)	β-Turn (%)	Random Coil (%)
0	16.20 ± 0.18 ^a^	40.74 ± 0.19 ^a^	26.56 ± 0.18 ^c^	16.50 ± 0.11 ^d^
0.1	15.89 ± 0.19 ^bc^	40.89 ± 0.12 ^a^	26.39 ± 0.17 ^c^	16.83 ± 0.13 ^c^
0.5	16.07 ± 0.17 ^ab^	39.58 ± 0.23 ^b^	27.47 ± 0.22 ^b^	16.88 ± 0.24 ^c^
1	15.71 ± 0.20 ^cd^	39.21 ± 0.16 ^c^	27.64 ± 0.11 ^b^	17.44 ± 0.17 ^b^
5	15.85 ± 0.14 ^bc^	38.69 ± 0.21 ^d^	28.04 ± 0.15 ^a^	17.42 ± 0.12 ^b^
10	15.48 ± 0.15 ^de^	37.93 ± 0.12 ^e^	28.22 ± 0.11 ^a^	18.37 ± 0.20 ^a^
15	15.39 ± 0.15 ^e^	37.95 ± 0.15 ^e^	28.20 ± 0.19 ^a^	18.46 ± 0.15 ^a^

All data points are represented as the mean of triplicate determinations ± standard errors. Values in the same column followed by different superscript letters (a–e) are significantly different (*p* < 0.05), which was concluded by SPSS software. In statistical analysis process, the effect of random error can be compared by probability value (*p*). If *p* < 0.05, it means that the effect of random error could be ignored and the difference of the values in the Table 2 are significative. Otherwise, the values in the same column followed by or within the same superscript letters are not significantly different (*p* > 0.05).

## Supplementary Materials

Supplementary Materials are available online. All data generated or analysed during this study are included in this published article and its supplementary information files.

## References

[1] Natarajan S., Luthria D., Bae H., Lakshman D., Mitra A. (2013). Transgenic Soybeans and Soybean Protein Analysis: An Overview. J. Agric. Food Chem..

[2] Moure A., Sineiro J., Dominguez H., Parajo J.C. (2006). Functionality of oilseed protein products: A review. Food Res. Int..

[3] Cavallieri A.L.F., Garcez M.M., Takeuchi K.P., da Cunha R.L. (2010). Heat-induced gels of soy protein and kappa-carrageenan at different pH values. Int. J. Food Sci. Technol..

[4] Kim K.M., Weller C.L., Hanna M.A., Gennadios A. (2002). Heat curing of soy protein films at selected temperatures and pressures. LWT—Food Sci. Technol..

[5] Harel S., Kanner J. (1985). Hydrogen peroxide generation in ground muscle tissues. J. Agric. Food Chem..

[6] Shacter E. (2000). Quantification and significance of protein oxidation in biological samples. Drug Metab. Rev..

[7] Davies M.J. (2005). The oxidative environment and protein damage. BBA Protein Proteom..

[8] Wu W., Zhang C.M., Kong X.Z., Hua Y.F. (2009). Oxidative modification of soy protein by peroxyl radicals. Food Chem..

[9] Maqsood S., Abushelaibi A., Manheem K., Rashedi A., Kadim I. (2015). Lipid oxidation, protein degradation, microbial and sensorial quality of camel meat as influenced by phenolic compounds. LWT—Food Sci. Technol..

[10] Zhou F., Zhao M., Cui C., Sun W. (2015). Influence of linoleic acid-induced oxidative modifications on physicochemical changes and in vitro digestibility of porcine myofibrillar proteins. LWT—Food Sci. Technol..

[11] Chanarat S., Benjakul S., Xiong Y.L. (2015). Physicochemical changes of myosin and gelling properties of washed tilapia mince as influenced by oxidative stress and microbial transglutaminase. J. Food Sci. Technol..

[12] Li Y.Q., Kong B.H., Xia X.F., Liu Q., Diao X.P. (2013). Structural changes of the myofibrillar proteins in common carp (Cyprinuscarpio) muscle exposed to a hydroxyl radical-generating system. Process Biochem..

[13] Liu Q., Lu Y., Han J.C., Chen Q., Kong B.H. (2015). Structure-modification by moderate oxidation in hydroxyl radical-generating systems promote the emulsifying properties of soy protein isolate. Food Struct..

[14] Kong B.H., Xiong Y.L., Cui X.H., Zhao X.H. (2013). Hydroxyl Radical-Stressed Whey Protein Isolate: Functional and Rheological Properties. Food Bioprocess Technol..

[15] Wu W., Hua Y.F., Lin Q.L., Xiao H.X. (2011). Effects of oxidative modification on thermal aggregation and gel properties of soy protein by peroxyl radicals. Int. J. Food Sci. Technol..

[16] Chen N., Zhao M., Sun W., Ren J., Cui C. (2013). Effect of oxidation on the emulsifying properties of soy protein isolate. Food Res. Int..

[17] Kinsella J.E. (1979). Functional properties of soy proteins. J. Am. Oil Chem. Soc..

[18] Fukushima D. (2001). Recent progress in research and technology on soybeans. Food Sci. Technol. Res..

[19] Hashidume T., Kato A., Tanaka T., Miyoshi S., Itoh N., Nakata R., Inoue H., Oikawa A., Nakai Y., Shimizu M. (2016). Single ingestion of soy β-conglycinin induces increased postprandial circulating FGF21 levels exerting beneficial health effects. Sci. Rep..

[20] Kimura A., Fukuda T., Zhang M., Motoyama S., Maruyama N., Utsumi S. (2008). Comparison of physicochemical properties of 7S and 11S globulins from pea, fava bean, cowpea, and French bean with those of soybean-French bean 7S globulin exhibits excellent properties. J. Agric. Food Chem..

[21] Meinlschmidt P., Schweiggert-Weisz U., Eisner P. (2016). Soy protein hydrolysates fermentation: Effect of debittering and degradation of major soy allergens. LWT—Food Sci. Technol..

[22] Uchida K., Kanematsu M., Sakai K., Matsuda T., Hattori N., Mizuno Y., Suzuki D., Miyata T., Noguchi N., Niki E. (1998). Protein-bound acrolein: Potential markers for oxidative stress. Proc. Natl. Acad. Sci. USA.

[23] Sante-Lhoutellier V., Aubry L., Gatellier P. (2007). Effect of oxidation on in vitro digestibility of skeletal muscle myofibrillar proteins. J. Agric. Food Chem..

[24] Stadtman E.R. (1990). Metal ion-catalyzed oxidation of proteins: Biochemical mechanism and biological consequences. Free Radic. Biol. Med..

[25] Zhang W.G., Xiao S., Ahn D.U. (2013). Protein Oxidation: Basic Principles and Implications for Meat Quality. Crit. Rev. Food Sci. Nutr..

[26] Wu W., Wu X.J., Hua Y.F. (2010). Structural modification of soy protein by the lipid peroxidation product acrolein. LWT—Food Sci. Technol..

[27] Ye L., Liao Y., Zhao M., Sun W. (2013). Effect of protein oxidation on the conformational properties of peanut protein isolate. J. Chem..

[28] Liu G., Xiong Y.L., Butterfield D.A. (2000). Chemical, Physical, and Gel-forming properties of oxidized myofibrils and whey- and soy-protein isolates. J. Food Sci..

[29] Kozarova A., Sliskovic I., Mutus B. (2007). Identification of redox sensitive thiols of protein disulfide isomerase using isotope coded affinity technology and mass spectrometry. J. Am. Soc. Mass Spectrom..

[30] Eaton P. (2006). Protein thiol oxidation in health and disease: Techniques for measuring disulfides and related modifications in complex protein mixtures. Free Radic. Biol. Med..

[31] Segat A., Misra N.N., Fabbro A., Buchini F., Lippe G., Cullen P.J., Innocente N. (2014). Effects of ozone processing on chemical, structural and functional properties of whey protein isolate. Food Res. Int..

[32] Wang Y.T., Wang Z.J., Handa C.L., Xu J. (2017). Effects of ultrasound pre-treatment on the structure of β-conglycinin and glycincin and the antioxidant activity of their hydrolysates. Food Chem..

[33] Chen L., Hackman R.M., Li C., Xu X., Zhou G., Feng X. (2016). Different physicochemical, structural and digestibility characteristics of myofibrillar protein from PSE and normal pork before and after oxidation. Meat Sci..

[34] Feng X., Li C., Ullah N., Cao J., Lan Y., Ge W., Hackman R.M., Li Z., Chen L. (2015). Susceptibility of whey protein isolate to oxidation and changes in physicochemical, structural, and digestibility characteristics. J. Dairy Sci..

[35] Wu W., Hou L., Zhang C.M., Kong X.Z., Hua Y.F. (2009). Structural modification of soy protein by 13-hydroperoxyoctadecadienoic acid. Eur. Food Res. Technol..

[36] Levine R.L., Berlett B.S., Moskovitz J., Mosoni L., Stadtman E.R. (1999). Methionine residues may protect proteins from critical oxidative damage. Mech. Ageing Dev..

[37] Wu W., Zhang C.M., Hua Y.F. (2009). Structural modification of soy protein by the lipid peroxidation product malondialdehyde. J. Sci. Food Agric..

[38] Tang C.H., Sun X., Foegeding E.A. (2011). Modulation of physicochemical and conformational properties of kidney bean vicilin (phaseolin) by glycation with glucose: Implications for structure–function relationships of legume vicilins. J. Agric. Food Chem..

[39] Jamdar S.N., Rajalakshmi V., Pednekar M.D., Juan F., Yardi V., Sharma A. (2010). Influence of degree of hydrolysis onfunctional properties, antioxidant activity and ACE inhibitoryactivity of peanut protein hydrolysate. Food Chem..

[40] Xiong Y.L., Agyare K.K., Addo K. (2008). Hydrolyzed wheat gluten suppresses transglutaminase-mediated gelation but improves emulsification of pork myofibrillar protein. Meat Sci..

[41] Nishinari K., Fang Y., Guo S., Phillips G.O. (2014). Soy proteins: A review on composition, aggregation and emulsification. Food Hydrocoll..

[42] Jonathan O.S., Jack B., Michael P., Richard G., Ian N. (2015). Comparative assessment of the effect of ultrasound treatment on protein functionality pre- and post-emulsification. Colloids Surf. A Physicochem. Eng. Asp..

[43] Cui X.H., Xiong Y.L., Kong B.H., Zhao X.H., Liu N. (2012). Hydroxyl radical-stressed whey protein isolate: Chemical and structural properties. Food Bioprocess Technol..

[44] L’Hocine L., Boye J.I., Munyana C. (2007). Detection and quantification of soy allergens in food: Study of two commercial enzyme-linked immunosorbent assays. J. Food Sci..

[45] Shreffler W.G., Beyer K., Chu T.T., Burks A.W., Sampson H.A. (2004). Microarray immunoassay: Association of clinical history, in vitro IgE function, and heterogeneity of allergenic peanut epitopes. J. Allergy Clin. Immunol..

[46] Krishnan H.B., Kim W., Jang S., Kerley M.S. (2009). All these subunits of soybean β-conglycinin are potential food allergens. J. Agric. Food Chem..

[47] Matsuo H., Yokooji T., Taogoshi T. (2015). Common food allergens and their IgE-binding epitopes. Allergol. Int..

[48] Song Y., Li Z., Lin H., Du S., Hao Z., Lin H., Zhu Z. (2015). Effect of malondialdehyde treatment on the IgE binding capacity and conformational structure of shrimp tropomyosin. Food Chem..

[49] Li Z., Lu Z., Khan M.N., Lin H., Zhang L. (2014). Protein carbonylation during electron beam irradiation may be responsible for changes in IgE binding to turbot parvalbumin. Food Chem. Toxicol..

[50] Benjakul S., Morrissey M.T. (1997). Protein Hydrolysates from Pacific Whiting Solid Wastes. J. Agric. Food Chem..

[51] Huang Y.R., Hua Y.F., Qiu A.Y. (2006). Soybean protein aggregation induced by lipoxygenase catalyzed linoleic acid oxidation. Food Res. Int..

[52] Pearce K.N., Kinsella J.E. (1978). Emulsifying properties of protein: Evaluation of turbidmetric technique. J. Agric. Food Chem..

[53] Laemmli U.K. (1970). Cleavage of structural proteins during the assembly of the head of bacteriophage T4. Nature.

[54] Wang Z., Li L., Yuan D., Zhao X., Cui S., Hu J., Wang J. (2014). Reduction of the allergenic protein in soybeanmeal by enzymatic hydrolysis. Food Agric. Immunol..

