# Supplementary material for: Structural and Functional Properties Changes of β-Conglycinin Exposed to Hydroxyl Radical-Generating Systems
Source: Molecules. 2017 Nov 3;22(11):1893. doi: 10.3390/molecules22111893 (PMC6150227; doi:10.3390/molecules22111893)
Supplement: Supplementary file 1 [file molecules-22-01893-s001.pdf]

Table.1. Changes in protein carbonyls

H_2O_2 (mmol/L)	Carbonyls (nmol/mg)		
0	7.92	7.79	8.35
0.1	8.19	7.96	8.28
0.5	8.10	8.29	8.65
1	9.09	8.99	9.47
5	10.89	11.15	10.78
10	13.64	14.01	13.55
15	14.38	14.66	14.32

Table.2. Changes in protein histidine

H_2O_2 (mmol/L)	Histidine (g/100g)		
0	1.17	1.16	1.17
0.1	1.18	1.16	1.14
0.5	1.17	1.15	1.14
1	1.14	1.13	1.13
5	1.12	1.10	1.09
10	1.11	1.09	1.05
15	1.05	1.04	1.04

Table.3. Changes in protein lysine

H_2O_2 (mmol/L)	Lysine (g/100g)		
0	2.90	2.93	2.91
0.1	2.86	2.92	2.90
0.5	2.79	2.84	2.83
1	2.76	2.81	2.80
5	2.69	2.73	2.72
10	2.58	2.66	2.63
15	2.51	2.57	2.54

Table.4. Changes in protein available lysine

H_2O_2 (mmol/L)	Available lysine (g/100g)		
0	6.17	6.21	6.32
0.1	5.89	5.94	6.04
0.5	5.31	5.42	5.59
1	5.16	4.94	5.09
5	4.22	4.48	4.17
10	4.08	4.15	4.03
15	3.72	3.79	3.54

Table.5. Changes in protein free SH

H_2O_2 (mmol/L)	Free SH (nmol/mg)		
0	8.62	8.69	8.67
0.1	8.58	8.59	8.58
0.5	7.40	7.45	7.41
1	5.17	5.20	5.17
5	1.36	1.32	1.41
10	1.06	0.94	1.04
15	0.96	0.91	0.99

Table.6. Changes in protein disulfide groups

H_2O_2 (mmol/L)	Disulfide groups (nmol/mg)		
0	85.39	86.88	82.40
0.1	84.99	80.34	78.94
0.5	77.38	80.89	79.48
1	84.84	89.00	91.49
5	85.50	89.71	92.19
10	95.29	93.82	96.44
15	99.63	93.25	95.85

Table.7. Changes in protein secondary structures (α–helix and β–sheet)

H_2O_2 (mmol/L)	α–helix (%)			β–sheet (%)		
0	16.04	16.04	16.4	40.91	40.53	40.78
0.1	15.71	15.71	16.08	41.02	40.77	40.89
0.5	15.92	15.92	16.25	39.52	39.83	39.4
1	15.51	15.51	15.91	39.05	39.24	39.35
5	15.73	15.73	16.01	38.93	38.51	38.63
10	15.36	15.36	15.65	37.81	38.05	37.93
15	15.26	15.26	15.55	38.09	37.96	37.8

Table.8. Changes in protein secondary structures (β–turn and Random coil)

H_2O_2 (mmol/L)	β–turn (%)			Random coil (%)		
0	26.62	26.36	26.71	16.46	16.41	16.63
0.1	26.57	26.23	26.38	16.98	16.71	16.81
0.5	27.71	27.28	27.43	16.97	16.61	17.07
1	27.77	27.55	27.61	17.6	17.27	17.44
5	28.21	27.99	27.92	17.54	17.42	17.30
10	28.10	28.32	28.25	18.59	18.21	18.31
15	27.99	28.34	28.27	18.48	18.61	18.30

Table.9. Changes in protein surface hydrophobicity

H_2O_2 (mmol/L)	Surface hydrophobicity (H_0)		
0	1977.5	1880.91	1922.62
0.1	1872.25	1899.8	1792.71
0.5	1853.57	1782.19	1802.13
1	1863.76	1754.58	1793.49
5	2025.04	1901.41	1966.81
10	2134.19	2191.45	2272.95
15	2404.37	2321.15	2249.07

Table.10. Changes in protein fluorescence intensity

Wavelength(nm)	0	0.1	0.5	1	5	10	15
300	22.09505	19.27913	15.84474	17.77913	14.41435	16.58825	20.52687
300.5	22.97402	20.02066	16.71267	18.52066	15.01992	17.46174	21.21948
301	23.83851	20.76567	17.52177	19.26567	15.51461	18.33317	21.99489
301.5	24.71571	21.4903	18.28314	19.9903	16.12561	19.21258	22.75658
302	25.61159	22.19662	19.09409	20.69662	17.001	20.08422	23.48371
302.5	26.5048	22.88663	19.9522	21.38663	17.1656	20.92755	24.18988
303	27.38048	23.58032	20.87125	22.08032	17.91338	21.74876	24.91418
303.5	28.23429	24.29093	21.7093	22.79093	18.61539	22.56007	25.64237
304	29.0981	25.00696	22.40369	23.50696	19.2316	23.36067	26.36538
304.5	30.0061	25.71996	22.99283	24.21996	19.9144	24.14641	27.0658
305	30.93367	26.41699	23.68253	24.91699	20.66313	24.92492	27.78933
305.5	31.84551	27.11104	24.50253	25.61104	21.22627	25.71794	28.52196
306	32.71855	27.77378	25.39065	26.27378	22.02018	26.51624	29.26743
306.5	33.59869	28.43963	26.24049	26.93963	22.73964	27.31841	29.9698
307	34.50798	29.14023	27.07771	27.64023	23.48609	28.09576	30.6378
307.5	35.44335	29.86909	27.92132	28.36909	24.11204	28.8972	31.2505
308	36.36109	30.59674	28.77162	29.09674	24.86238	29.73645	31.85486
308.5	37.23218	31.31433	29.60887	29.81433	25.64474	30.61611	32.47549
309	38.07788	32.06669	30.40537	30.56669	26.47901	31.46808	33.14031
309.5	38.95473	32.85081	31.17828	31.35081	27.31519	32.29331	33.8426
310	39.90439	33.65865	31.94933	32.15865	28.10917	33.08177	34.56359
310.5	40.86286	34.45152	32.74621	32.95152	28.89295	33.86399	35.22854

311	41.76499	35.23175	33.54374	33.73175	29.6868	34.61394	35.91579
311.5	42.60043	35.99356	34.32382	34.49356	30.57354	35.33924	36.70591
312	43.46109	36.77964	35.09911	35.27964	31.42298	36.05339	37.6467
312.5	44.39384	37.60784	35.94134	36.10784	32.24671	36.79098	38.57976
313	45.40609	38.46286	36.83472	36.96286	33.08212	37.55526	39.43184
313.5	46.42049	39.30856	37.71504	37.80856	33.84563	38.35524	40.19459
314	47.41255	40.11407	38.5132	38.61407	34.52612	39.21583	40.97253
314.5	48.35377	40.90858	39.29213	39.40858	35.19764	40.14236	41.74828
315	49.36562	41.71624	40.10745	40.21624	35.9981	41.03803	42.54548
315.5	50.37552	42.55629	40.97138	41.05629	36.89967	41.85966	43.31036
316	51.40643	43.39043	41.85571	41.89043	37.81255	42.64413	44.03321
316.5	52.46405	44.51575	42.6733	42.71575	38.6825	43.46891	44.73034
317	53.53047	45.5515	43.38738	43.5215	39.52954	44.32311	45.52464
317.5	54.57862	46.5186	44.0587	44.3186	40.38516	45.17631	46.41827
318	55.59117	47.26637	44.834	45.06637	41.24093	46.02307	47.37563
318.5	56.5977	47.98654	45.72849	45.78654	42.13717	46.89876	48.31527
319	57.59194	48.74496	46.69114	46.54496	43.06744	47.766	49.27622
319.5	58.57445	49.60856	47.66699	47.40856	44.02753	48.61436	50.24953
320	59.54126	50.54884	48.68081	48.34884	44.99189	49.42496	51.21696
320.5	60.52974	51.49442	49.69912	49.29442	45.95034	50.22302	52.16997
321	61.51817	52.40993	50.69154	50.20993	46.89019	51.00367	53.13623
321.5	62.5068	53.32494	51.68492	51.12494	47.75835	51.79	54.15035
322	63.4852	54.24323	52.70073	52.04323	48.65717	52.59112	55.18
322.5	64.50066	55.17096	53.73736	52.97096	49.64259	53.4428	56.20144
323	65.52515	56.1023	54.76206	53.9023	50.76211	54.33819	57.18985
323.5	66.54834	57.05982	55.72966	54.85982	51.87191	55.34014	58.15184
324	67.51998	58.02008	56.63414	55.82008	52.9369	56.44238	59.12908
324.5	68.49035	58.97068	57.54735	56.77068	53.9578	57.56484	60.06532
325	69.45684	59.92768	58.52327	57.72768	55.00904	58.58831	60.95461
325.5	70.45048	60.89976	59.56409	58.69976	56.081	59.42185	61.81473
326	71.44046	61.89352	60.6211	59.69352	57.1654	60.31025	62.76606
326.5	72.42819	62.88883	61.68005	60.68883	58.24276	60.94634	63.80581
327	73.38162	63.90644	62.74021	61.70644	59.30758	61.68653	64.92667
327.5	74.32838	64.94336	63.78161	62.74336	60.36866	62.4906	66.04581
328	75.30832	65.99593	64.79389	63.79593	61.43891	63.29998	67.14686
328.5	76.35528	67.02418	65.75421	64.82418	62.50593	64.13367	67.94555
329	77.09495	67.50517	66.26139	65.30517	63.09243	64.99287	68.59199
329.5	77.5796	67.86362	66.70013	65.66362	63.54837	65.59964	69.2232
330	77.98841	68.25296	67.22848	66.05296	63.98438	66.48934	69.76567
330.5	78.34114	68.6843	67.6869	66.4843	64.36809	67.07414	70.17594
331	78.65546	69.07835	68.09487	66.87835	64.72446	67.66594	70.64175
331.5	78.97064	69.44493	68.46086	67.24493	65.08833	68.05883	71.25989
332	79.30616	69.77034	68.84614	67.57034	65.43127	68.58936	71.57301
332.5	79.64315	70.0692	69.16273	67.8692	65.71625	68.97018	71.82006

333	79.92626	70.35049	69.40476	68.15049	65.96289	69.31113	72.0376
333.5	80.13829	70.62993	69.63533	68.42993	66.24108	69.68627	72.27774
334	80.35945	70.91953	69.91964	68.71953	66.54568	69.95863	72.6097
334.5	80.62323	71.18729	70.26152	68.98729	66.81579	70.13616	72.97431
335	80.9186	71.45464	70.54842	69.25464	67.03137	70.57492	73.3168
335.5	81.14344	71.70071	70.76026	69.50071	67.22265	70.84471	73.49476
336	81.32603	71.93473	70.89478	69.73473	67.36456	71.04771	73.64444
336.5	81.48976	72.15926	71.04173	69.95926	67.48965	71.26633	73.91879
337	81.69181	72.37973	71.17142	70.17973	67.61032	71.49085	74.10318
337.5	81.88865	72.56612	71.28451	70.36612	67.69988	71.55628	74.26551
338	82.0284	72.68713	71.32019	70.48713	67.7903	71.62094	74.45756
338.5	82.07742	72.74542	71.36094	70.54542	67.87873	71.7315	74.56762
339	82.08462	72.75815	71.39703	70.55815	67.94396	71.83212	74.76547
339.5	82.10379	72.76741	71.48233	70.56741	68.01535	71.9191	74.84957
340	82.12585	72.76899	71.51405	70.56899	68.09848	71.9482	74.92861
340.5	82.10415	72.74816	71.47185	70.54816	68.15078	71.99744	75.01049
341	82.00477	72.69144	71.43566	70.49144	68.27976	72.00209	75.04483
341.5	81.88129	72.59286	71.35199	70.39286	68.35818	72.01325	75.08334
342	81.75749	72.47376	71.29409	70.27376	68.46101	72.02069	75.10161
342.5	81.61871	72.35463	71.24901	70.15463	68.45125	72.06707	75.12615
343	81.43421	72.26677	71.20883	70.06677	68.40163	71.98376	75.14841
343.5	81.24343	72.21905	71.15528	70.01905	68.33449	71.9024	75.15914
344	81.05944	72.17727	71.08506	69.97727	68.2689	71.85715	75.06473
344.5	80.89026	72.08932	70.90051	69.88932	68.20264	71.53871	74.93639
345	80.71064	71.92335	70.78249	69.72335	68.16069	71.33655	74.78386
345.5	80.45865	71.72022	70.69904	69.52022	68.03167	71.13625	74.65021
346	80.12426	71.5216	70.5761	69.3216	67.94025	70.86594	74.44436
346.5	79.75597	71.34872	70.44953	69.14872	67.83456	70.59922	74.1962
347	79.47981	71.13097	70.26335	68.93097	67.70902	70.18041	73.90696
347.5	79.27285	70.86905	70.01785	68.66905	67.53118	69.79763	73.57681
348	79.07725	70.57193	69.74553	68.37193	67.35456	69.5208	73.21346
348.5	78.83401	70.27124	69.48885	68.07124	67.21644	69.26508	72.86038
349	78.58812	69.9321	69.26884	67.7321	67.03494	69.02232	72.51371
349.5	78.2989	69.58011	69.09068	67.38011	66.87345	68.78973	72.10857
350	77.93837	69.25441	68.9207	67.05441	66.66079	68.47238	71.68753
350.5	77.51859	68.95622	68.7124	66.75622	66.37861	68.09867	71.30631
351	77.08894	68.62403	68.45456	66.42403	66.0412	67.67107	71.00111
351.5	76.69403	68.23529	68.15645	66.03529	65.65704	67.29765	70.62005
352	76.32359	67.81159	67.82287	65.61159	65.26991	66.98516	70.13065
352.5	75.96651	67.36667	67.44499	65.16667	64.87534	66.70179	69.54388
353	75.58263	66.22569	67.03478	64.72569	64.47651	66.37093	68.9785
353.5	75.16515	65.80951	66.61371	64.30951	64.04156	65.9989	68.41475
354	74.70586	65.44195	66.17716	63.94195	63.56407	65.62578	67.86159
354.5	74.20357	65.1014	65.72104	63.6014	63.04307	65.30132	67.3175

355	73.67583	64.70044	65.2858	63.20044	62.53951	64.98784	66.76582
355.5	73.15576	64.21402	64.88973	62.71402	62.00475	64.59847	66.20027
356	72.66808	63.64709	64.51595	62.14709	61.40444	64.11555	65.61196
356.5	72.21401	63.07779	64.08259	61.57779	60.74842	63.62849	65.00674
357	71.76555	62.51869	63.57392	61.01869	60.1643	63.20741	64.37533
357.5	71.25365	61.97221	63.00963	60.47221	59.66103	62.8529	63.71958
358	70.70878	61.42797	62.4529	59.92797	59.19756	62.47409	63.1214
358.5	70.17343	60.92838	61.92567	59.42838	58.69351	62.02055	62.60255
359	69.67522	60.47614	61.4292	58.97614	58.15468	61.50828	62.11496
359.5	69.13275	60.01846	60.91097	58.51846	57.59332	61.00944	61.56289
360	68.54226	59.51721	60.33613	58.01721	57.05754	60.60313	60.96539
360.5	67.90077	58.98222	59.72813	57.48222	56.58479	60.231	60.34192
361	67.27727	58.4797	59.11044	56.9797	56.11674	59.82579	59.73455
361.5	66.63366	58.01387	58.5255	56.51387	55.58675	59.37019	59.15409
362	65.995	57.53573	57.95907	56.03573	54.97703	58.901	58.59057
362.5	65.33716	57.01959	57.4492	55.51959	54.36886	58.41167	57.9942
363	64.6613	56.48959	56.93978	54.98959	53.81645	57.91643	57.32504
363.5	63.96702	55.96556	56.38729	54.46556	53.32678	57.40974	56.64421
364	63.26573	55.43989	55.76336	53.93989	52.79246	56.915	56.01126
364.5	62.58336	54.9092	55.14195	53.4092	52.29048	56.40537	55.44639
365	61.89861	54.37933	54.56742	52.87933	51.89067	55.85925	54.92606
365.5	61.23182	53.85481	54.01065	52.35481	51.6161	55.30414	54.44533
366	60.54761	53.32977	53.45425	51.82977	51.28049	54.76907	53.97133
366.5	59.9024	52.82415	52.89864	51.32415	50.86508	54.26947	53.49175
367	59.28545	52.37389	52.36119	50.87389	50.42426	53.78095	53.01452
367.5	58.70894	51.99722	51.80471	50.49722	50.04824	53.29164	52.56243
368	58.12343	51.56307	51.19922	50.06307	49.67438	52.78238	52.12389
368.5	57.56293	51.00774	50.6337	49.50774	49.29724	52.26563	51.68173
369	57.00347	50.35946	50.16514	48.85946	48.94509	51.73489	51.2236
369.5	56.44375	49.81972	49.80518	48.31972	48.60338	51.20578	50.73117
370	55.86717	49.41393	49.41233	47.91393	48.22729	50.68841	50.22006
370.5	55.28374	49.09602	48.98327	47.59602	47.79469	50.1821	49.71625
371	54.74803	48.7338	48.49396	47.2338	47.2977	49.65555	49.26773
371.5	54.27211	48.35797	48.03378	46.85797	46.76022	49.06933	48.85958
372	53.8623	47.98195	47.60489	46.48195	46.22202	48.49257	48.46825
372.5	53.43466	47.6492	47.24288	46.1492	45.74171	48.02818	48.06802
373	52.99514	47.32772	46.9188	45.82772	45.34753	47.68345	47.66075
373.5	52.51088	47.00204	46.58733	45.50204	45.00433	47.32737	47.24827
374	52.01817	46.65721	46.22478	45.15721	44.6401	46.83702	46.81919
374.5	51.4636	46.31108	45.84121	44.81108	44.19892	46.22824	46.38451
375	50.936	45.94224	45.43408	44.44224	43.71892	45.55493	45.96557
375.5	50.46275	45.54364	45.00241	44.04364	43.22791	44.95376	45.57885
376	50.05192	45.12173	44.56823	43.62173	42.77531	44.46558	45.20148
376.5	49.63668	44.70869	44.1517	43.20869	42.29482	44.09768	44.80338

377	49.22165	44.30864	43.73155	42.80864	41.81041	43.68317	44.41198
377.5	48.80461	43.92904	43.28671	42.42904	41.3235	43.21709	44.04275
378	48.38855	43.5707	42.82451	42.0707	40.86241	42.66256	43.66709
378.5	47.96004	41.37591	42.39967	41.70879	40.37087	42.03442	43.22258
379	47.5232	40.8962	41.99333	41.29364	39.84391	41.31574	42.74863
379.5	47.09184	40.41227	41.57592	40.81459	39.32243	40.64378	42.30482
380	46.67782	39.93978	41.11333	40.3099	38.8299	40.09525	41.9095
380.5	46.29191	39.45646	40.61944	39.8283	38.29258	39.71182	41.44124
381	45.90798	38.94149	40.09317	39.37586	37.76222	39.41914	40.85882
381.5	45.50446	38.41401	39.56343	38.93663	37.32853	39.16748	40.22957
382	45.05768	37.92933	39.03864	38.48542	37.00497	38.87748	39.69547
382.5	44.58211	37.49047	38.54398	38.03361	36.63174	38.53033	39.2649
383	44.09406	37.04174	38.07034	37.60893	36.16225	38.14374	38.87436
383.5	43.5841	36.53305	37.61795	37.2052	35.65509	37.76767	38.46434
384	43.03262	35.9658	37.17261	36.77823	35.20558	37.40234	38.06808
384.5	42.45784	35.39779	36.70009	36.28868	34.76612	37.02995	37.69659
385	41.89478	34.85733	36.18373	35.77418	34.31592	36.62902	37.31274
385.5	41.35141	34.35912	35.65504	35.28563	33.85279	36.17683	36.88829
386	40.80836	33.87255	35.15465	34.8119	33.37865	35.71339	36.42952
386.5	40.24089	33.37596	34.69156	34.29507	32.89795	35.2494	35.96299
387	39.66475	32.83918	34.22159	33.71536	32.42555	34.78101	35.48497
387.5	39.0823	32.27022	33.71659	33.1227	31.96661	34.3118	34.9818
388	38.51165	31.70423	33.16519	32.54806	31.5204	33.86927	34.45753
388.5	37.97968	31.16533	32.63404	31.98447	31.07176	33.43187	33.92158
389	37.49643	30.66859	32.14291	31.39167	30.62947	32.95483	33.38992
389.5	37.04058	30.1856	31.69886	30.78557	30.2066	32.42657	32.87161
390	36.54601	29.7213	31.22705	30.20299	29.80077	31.89045	32.36791
390.5	35.99127	29.24401	30.70806	29.66247	29.38427	31.38767	31.84348
391	35.39537	28.76872	30.16101	29.14293	28.94456	30.91795	31.27802
391.5	34.83918	28.29893	29.65424	28.62733	28.51188	30.4813	30.6996
392	34.30776	27.85597	29.18663	28.1076	28.10178	30.05859	30.1547
392.5	33.77904	27.40741	28.72316	27.57252	27.71063	29.62876	29.64653
393	33.22119	26.93771	28.22003	26.98654	27.29361	29.15352	29.15485
393.5	32.67539	26.45431	27.69255	26.44892	26.84205	28.67506	28.65322
394	32.12228	25.98768	27.18179	26.03907	26.35387	28.14094	28.16465
394.5	31.56847	25.5644	26.72328	25.77414	25.85556	27.55656	27.69679
395	31.00697	25.18183	26.2913	25.49978	25.36165	26.94232	27.26166
395.5	30.45711	24.83194	25.87119	25.16296	24.90161	26.42156	26.83007
396	29.90854	24.49673	25.44795	24.77227	24.46525	25.98423	26.39828
396.5	29.38226	24.16407	25.05489	24.41056	24.07584	25.5934	25.9583
397	28.89081	23.812	24.69003	24.05714	23.72716	25.17529	25.53022
397.5	28.43941	23.44748	24.32996	23.6956	23.40336	24.77138	25.10806
398	27.99564	23.07743	23.9491	23.31823	23.05269	24.36362	24.69566
398.5	27.52969	22.72015	23.558	22.93999	22.66036	23.93398	24.2981

399	27.0573	22.3779	23.18635	22.57012	22.26042	23.46675	23.91052
399.5	26.58665	22.06668	22.83253	22.21028	21.90441	22.99301	23.53596
400	26.15079	21.79241	22.4857	21.85557	21.59583	22.52658	23.17069
400.5	25.72237	21.54218	22.13538	21.51564	21.29558	22.08268	22.81795
401	25.30796	21.27272	21.79826	21.18655	20.97893	21.65112	22.45287
401.5	24.90494	20.98521	21.47203	20.86425	20.65129	21.25182	22.07281
402	24.54567	20.69989	21.13947	20.54172	20.3509	20.88171	21.68534
402.5	24.17129	20.43194	20.80802	20.22711	20.07158	20.53569	21.29516
403	23.77007	20.17444	20.50381	19.91267	19.79423	20.14456	20.89853
403.5	23.3691	19.9212	20.24536	19.60604	19.5006	19.75439	20.51235
404	23.0187	19.67045	19.99452	19.31659	19.21726	19.48067	20.15876
404.5	22.70766	19.43013	19.72831	19.05761	18.95858	19.40622	19.83215
405	22.39011	19.19704	19.44717	18.81775	18.72056	19.4077	19.50605
405.5	22.06873	18.9712	19.18243	18.59731	18.48405	19.33288	19.13778
406	21.75814	18.76137	18.94203	18.39479	18.2739	19.12828	18.82547
406.5	21.47256	18.56958	18.72797	18.22052	18.0791	18.89964	18.63414
407	21.17499	18.38081	18.53546	18.05946	17.88497	18.70644	18.55197
407.5	20.86986	18.16293	18.3599	17.90802	17.66593	18.5365	18.4412
408	20.55339	17.90705	18.18771	17.76294	17.45192	18.3643	18.26767
408.5	20.25502	17.64226	18.01602	17.60982	17.26129	18.17515	18.07104
409	19.96566	17.40658	17.83793	17.43659	17.08436	17.98094	17.90308
409.5	19.6999	17.21083	17.66586	17.24254	16.90247	17.78492	17.72247
410	19.4584	17.04457	17.50611	17.06581	16.73096	17.6146	17.51578
410.5	19.24683	16.89216	17.36958	16.90726	16.57908	17.459	17.3163
411	19.0452	16.73366	17.22715	16.75232	16.44453	17.31245	17.12845
411.5	18.85387	16.57176	17.07087	16.59053	16.30492	17.12988	16.94845
412	18.67568	16.42207	16.90907	16.44056	16.16885	16.93527	16.77068
412.5	18.50421	16.30175	16.77552	16.31143	16.05164	16.74765	16.58649
413	18.33453	16.20153	16.65674	16.18544	15.96702	16.58231	16.40294
413.5	18.1703	16.09769	16.53607	16.05516	15.89173	16.40919	16.22712
414	18.00988	15.98324	16.39131	15.92316	15.81199	16.22381	16.08048
414.5	17.83545	15.85837	16.23931	15.80952	15.71118	16.05622	15.95619
415	17.64125	15.73792	16.08818	15.70752	15.60151	15.91477	15.84396
415.5	17.45214	15.62562	15.95798	15.60862	15.48532	15.80311	15.72237
416	17.29141	15.55795	15.84594	15.53292	15.44773	15.7658	15.59975
416.5	17.14372	15.45973	15.75644	15.43234	15.32299	15.61876	15.5102
417	16.98312	15.23796	15.6635	15.24061	15.17385	15.49931	15.40841
417.5	16.80421	14.86146	15.54177	14.91457	15.01499	15.2555	15.21073
418	16.61728	14.40044	15.36999	14.48404	14.8664	15.04291	14.86593
418.5	16.47677	14.36997	15.26067	14.45489	14.98855	14.72292	14.38453
419	16.34224	14.35492	15.15499	14.43947	14.7496	14.64353	14.35923
419.5	16.21367	14.35529	15.05295	14.43777	14.54955	14.40474	14.35047
420	16.09107	14.37107	14.95454	14.44979	14.5884	14.50655	14.35825

Table.11. Changes in protein water solubility

H_2O_2 (mmol/L)	Water solubility (g/100g)		
0	112.29	117.51	114.40
0.1	89.28	87.14	89.96
0.5	89.14	88.86	89.97
1	100.25	102.75	103.31
5	91.36	94.97	95.81
10	86.36	88.03	89.14
15	78.31	78.31	79.97

Table.12. Changes in protein EAI and ESI

H_2O_2 (mmol/L)	EAI (m^2/g)			ESI (min)		
0	28.98	27.89	26.46	41.86	40.24	42.9
0.1	29.31	27.87	27.33	41.97	44.1	44.07
0.5	27.63	29.24	29.59	45.29	43.34	44.35
1	29.74	30.98	31.35	41.93	45.12	43.79
5	32.3	31.9	34.38	44.9	44.1	46.02
10	34.26	33.85	36.08	44.34	46.44	44.05
15	36.38	35.17	38.31	48.58	47.27	49.76

Table.13. Changes in protein allergens content

H_2O_2 (mmol/L)	Allergens content (ug/g)		
0	17.82	19.05	17
0.1	17.05	16.16	15.37
0.5	15.48	16.63	14.88
1	14.99	14.82	13.37
5	12.57	10.66	11.99
10	5.7	4.78	5.55
15	5.17	4.47	5.47

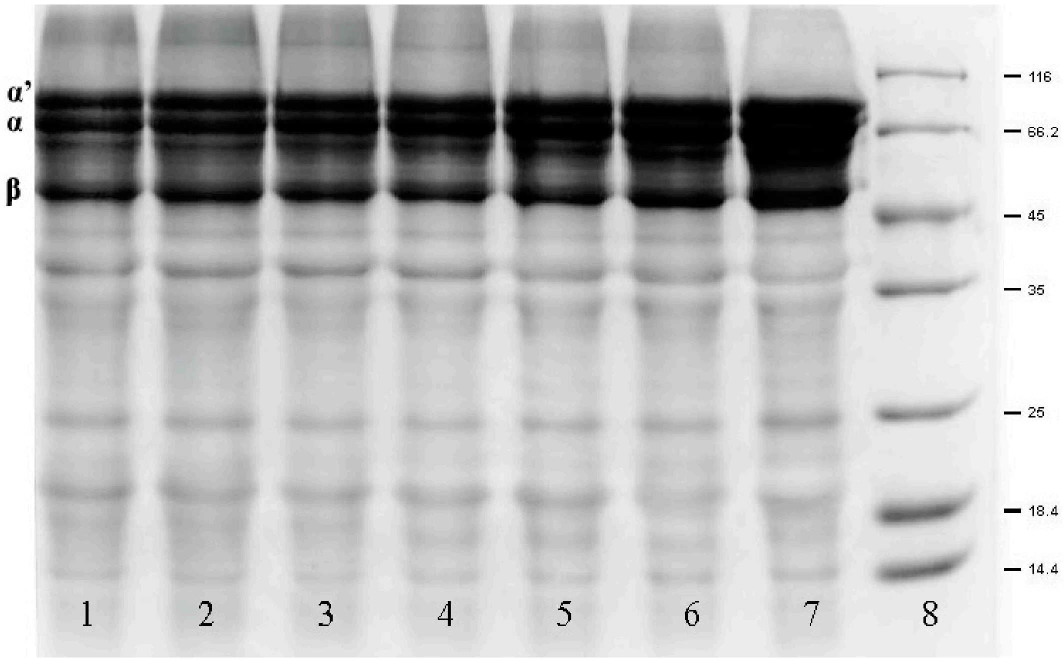

Fig. SDS–PAGE patterns of low molecular weight marker and oxidized β–conglycinin treated with different concentrations of H_2O_2 for 3h at 20 °C. α, α', and β bands showed the changes in three subunits of β–conglycinin. (Lane 1, 15 mmol/L H_2O_2; Lane 2, 10 mmol/L H_2O_2; Lane 3, 5 mmol/L H_2O_2; Lane 4, 1 mmol/LH_2O_2; Lane 5, 0.5 mmol/L H_2O_2; Lane 6, 0.1 mmol/L H_2O_2;

Lane 7, β–conglycinin; Lane 8, low molecular weight marker) The molecular weights of markers are 14.4 kDa, 18.4 kDa, 25.0 kDa, 35.0 kDa, 45.0 kDa, 66.2 kDa, and 116.0 kDa, respectively.
